# Implementation of Syndromic Surveillance Systems in Two Rural Villages in Senegal

**DOI:** 10.1371/journal.pntd.0005212

**Published:** 2016-12-07

**Authors:** Cédric Abat, Philippe Colson, Hervé Chaudet, Jean-Marc Rolain, Hubert Bassene, Aldiouma Diallo, Oleg Mediannikov, Florence Fenollar, Didier Raoult, Cheikh Sokhna

**Affiliations:** 1 Aix-Marseille Univ., URMITE UM 63 CNRS 7278 IRD 198 INSERM U1095, IHU Méditerranée Infection, Facultés de Médecine et de Pharmacie, 27 boulevard Jean Moulin, Marseille CEDEX 05, France; 2 Campus IRD d' Hann Maristes, Dakar, Sénégal; University of California San Diego School of Medicine, UNITED STATES

## Abstract

Infectious diseases still represent a major challenge for humanity. In this context, their surveillance is critical. From 2010 to 2016, two Point-Of-Care (POC) laboratories have been successfully implemented in the rural Saloum region of Senegal. In parallel, a homemade syndromic surveillance system called EPIMIC was implemented to monitor infectious diseases using data produced by the POC laboratory of the Timone hospital in Marseille, France. The aim of this study is to describe the steps necessary for implementing EPIMIC using data routinely produced by two POC laboratories (POC-L) established in rural Senegal villages. After improving EPIMIC, we started to monitor the 15 pathogens routinely diagnosed in the two POC-L using the same methodology we used in France. In 5 years, 2,577 deduplicated patients-samples couples from 775 different patients have been tested in the Dielmo and Ndiop POC-L. 739 deduplicated patients-samples couples were found to be positive to at least one of the tested pathogens. The retrospective analysis of the Dielmo and Ndiop POC data with EPIMIC allowed to generate 443 alarms. Since January 2016, 316 deduplicated patients-samples couples collected from 298 different patients were processed in the Niakhar POC laboratory. 56 deduplicated patients-samples couples were found to be positive to at least one of the tested pathogens. The retrospective analysis of the data of the Niakhar POC laboratory with EPIMIC allowed to generate 14 alarms. Although some improvements are still needed, EPIMIC has been successfully spread using data routinely produced by two rural POC-L in Senegal, West Africa.

## Author Summary

Infectious diseases are still a major public health problem for humanity, especially because of their unpredictability. In this context, developing surveillance systems is one of the most effective solutions to fight them, including in Sub-Saharan African countries where the situation is particularly worrying. This study describes how automated surveillance systems have been implemented in two rural areas in Senegal for the weekly surveillance of 15 pathogens responsible for fever in Africa and on the basis of laboratory data from two rural laboratories. In addition, the first results of this surveillance are presented. The two surveillance systems prospectively and retrospectively detected several abnormal events, and allowed us to observe that the distribution of the pathogens was not the same depending on the region under surveillance. This study provides evidence on the possibility of developing surveillance systems in rural areas in Africa. Such initiatives are needed, especially to improve our global knowledge on infectious diseases in this continent.

## Introduction

In their 2015 report, the Global Burden of Disease study group estimated that in 2013 54.9 million people died worldwide, with 11.8 million deaths due to communicable, maternal, neonatal, and nutritional disorders [1]. Infectious diseases were directly involved in a significant part of them, with 2.7 million deaths due to lower respiratory infections, 1.3 million deaths due to HIV/AIDS, 1.3 million deaths due to tuberculosis, 1.3 million deaths due to diarrhoeal diseases, and 854,600 deaths due to malaria [1]. This situation clearly underlines that infectious diseases are still a big challenge for humanity in the 21^st^ century, especially because i) we still discover more and more possible pathogens with new technologies, ii) pathogens are naturally evolving, leading to the emergence or re-emergence of pathogens, iii) people and resources are moving and exchanging faster and faster around the world, which directly affects the ecosystems, and iv) their appearance and disappearance in the different human populations cannot be reliably modeled [2–5].

Facing this situation, various strategies have been developed worldwide and over the time to try to monitor infectious diseases and pathogens. In this way, three main strategies of surveillance are currently extensively used for the monitoring of infectious diseases [6]: i) disease-specific surveillance allowing the surveillance of pathogens, syndromes or risk exposures defined to be public health threats in a precise population, ii) syndromic surveillance using non specific indicators not collected for surveillance purposes to be used for the early real-time identification of the impact (or non impact) of possible health threats, and iii) event-based surveillance using unstructured information from the Internet for the real-time or near real-time detection of potential or confirmed health events occuring in the world. Moreover, thirteen main sources of data are currently available for the infectious disease surveillance, including, among others, the Internet, drug sales reports, sentinel surveillance notifications, notifiable diseases reports, and microbiology orders reports.

The current and past impact of infectious diseases on humanity have also promotted the developpement and spread of new microbiology technologies and laboratories. Among the most innovative improvements made can be particularly mentioned Point-Of-Care (POC) laboratories (POC-L). They consist in on-site around the clock operating laboratories equipped to perform a wide variety of rapid diagnostic tests that are able to deliver rapid diagnosis [7,8]. Therefore, they can help to take adequate decisions for hospitalization, isolation and therapy in only few hours [7]. Moreover, these laboratories do not require higly specialized skills and equipments, making them potentially cheaper and more cost-effective than conventional laboratories, but equally easier to implement in place with low resources [8].

Sub-Saharan Africa is a 24 million km^2^ area that includes more than 1 billion people from 48 different countries (http://data.worldbank.org/). Although the situation is improving, with large gains in life expectancy mainly due to reductions of diarrhoea and lower respiratory infections, HIV/AIDS, malaria and tuberculosis still remained important cause of deaths in this area, especially in the southern, western and eastern sub-Saharan African countries [1]. Moreover, while the number of child deaths decreased significantly all around the world from 1990 to 2013, this number has only slightly decreased from 3,7 million (3,6–3,7) in 1990 to 3,2 million (3–3,4) in 2013 in the sub-Saharan Africa area, especially because of malaria, diarrhoeal diseases, lower respiratory infections, HIV/AIDS and measles [1].

From 2010 to 2016, two POC-L have been successfully implemented in the rural Saloum region of Senegal [9]. Using our 15-years experience of infectious diseases surveillance in the Assistance Publique- Hôpitaux de Marseille (AP-HM) institution, we recently decided to implement our homemade syndromic surveillance system EPIMIC (for EPIdemiological surveillance and alert based on MICrobiological data) [10] in Senegal to monitor infectious diseases diagnosed in the two rural POC-L using the data routinely produced by these laboratories. The aim of this study is to describe the implementation ad results of EPIMIC in a developing country using data routinely produced by two POC-L established in rural Senegal villages.

## Materials and Methods

### The study sites

**Fig 1:**
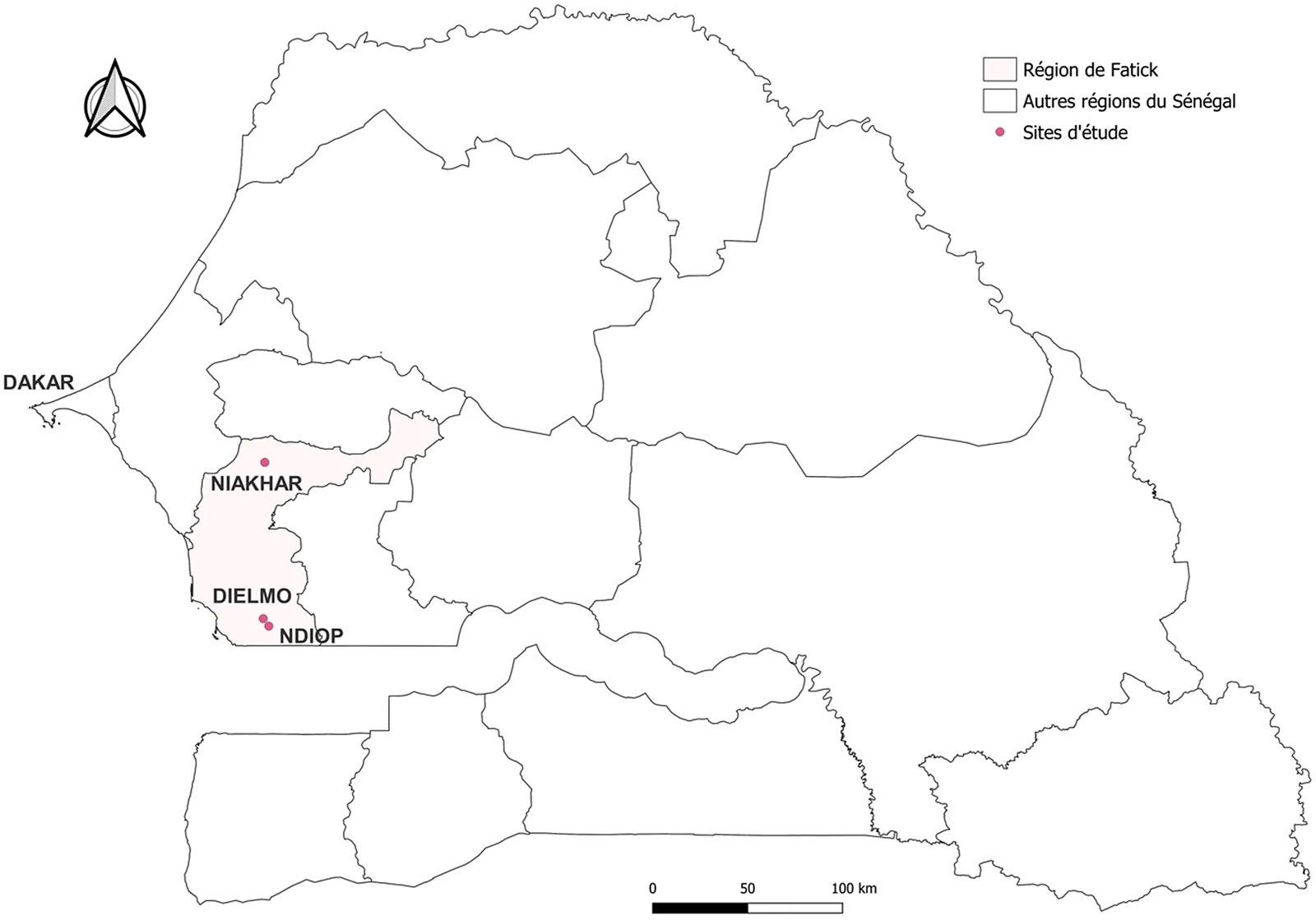
Geographic distribution of the two Point-Of-Care laboratories, Senegal.

The two POC-L have been implemented in two different sites (Fig 1), one located in the research station of Dielmo and deserving two rural villages in the Sine-Saloum region (Dielmo and Ndiop), and the other in the research station of the rural village of Niakhar. The first POC laboratory has been already extensively described elsewhere [9]. The second study site is located in the Fatick region of Senegal 155km South-East of Dakar. This site theoretically involves a ~48 000 inhabitants distributed in 30 villages covering a 230km^2^ area.

### The two POC laboratories

The functioning of the POC laboratory of Dielmo/Ndiop is similar to that previously extensively described by Sokhna et al. [9], and is now equipped with a reliable Internet connexion. For the Niakhar POC laboratory, the way of functioning is different although its equipment is similar to that of Dielmo/Ndiop [9]. Thus, as the area and the population covered by this POC laboratory is far more important than that of Dielmo/Ndiop villages, this POC laboratory is working in close collaboration with 4 health structures (Niakhar, Diohine, Toucar and Ngayokhem) located in some of the biggest villages of the area (Fig 1). To sum up, 5 days on 7, the technician from the POC laboratory visits all the health strucutures in motorcyle to collect in iceboxes the samples collected by the nurses from the patients visiting the 4 different health structures mentioned above. Then, the samples are tested by the technician in the POC laboratory to look for the possible causative agent responsible for the illness of the different patients. When the tests are performed, the technician completes a preformated Microsoft Excel worksheet summarizing the main information required to properly identify each patient. The same procedure is followed in the Dielmo/Ndiop POC laboratory. The information collected in the two POC-L are structurally similar (Table 1). The POC laboratory of Niakhar is also equipped with a reliable Internet connexion.

**Table 1:** Global features of the sites of Dielmo and Ndiop, and Niakhar.

	Sites	
	Dielmo and Ndiop	Niakhar
**Location**	280 km South-East of Dakar	155 km South-East of Dakar
**Number of villages**	2 villages	30 villages
**Population**	~750 inhabitants	~48 000 inhabitants

### The EPIMIC infectious diseases surveillance system

EPIMIC has been previously extensively described elsewhere [10]. Briefly, EPIMIC is a Microsoft Excel based syndromic surveillance system allowing the weekly automated surveillance of a large panel of pathogens. Thus, it allows the real-time monitoring of the number of samples tested and positives performed every week. The percentage of positivity for each test performed is also automatically calculated. The new values entered in the software are then automatically compared to their historical mean number +/- two standard deviations (thresholds), and alarms are automatically emitted by EPIMIC when the last entered values (the number of tested samples, the number of positive samples and the percentage of positiveness) exceed the thresholds. Plots are then automatically produced by the software.

**Fig 2:**
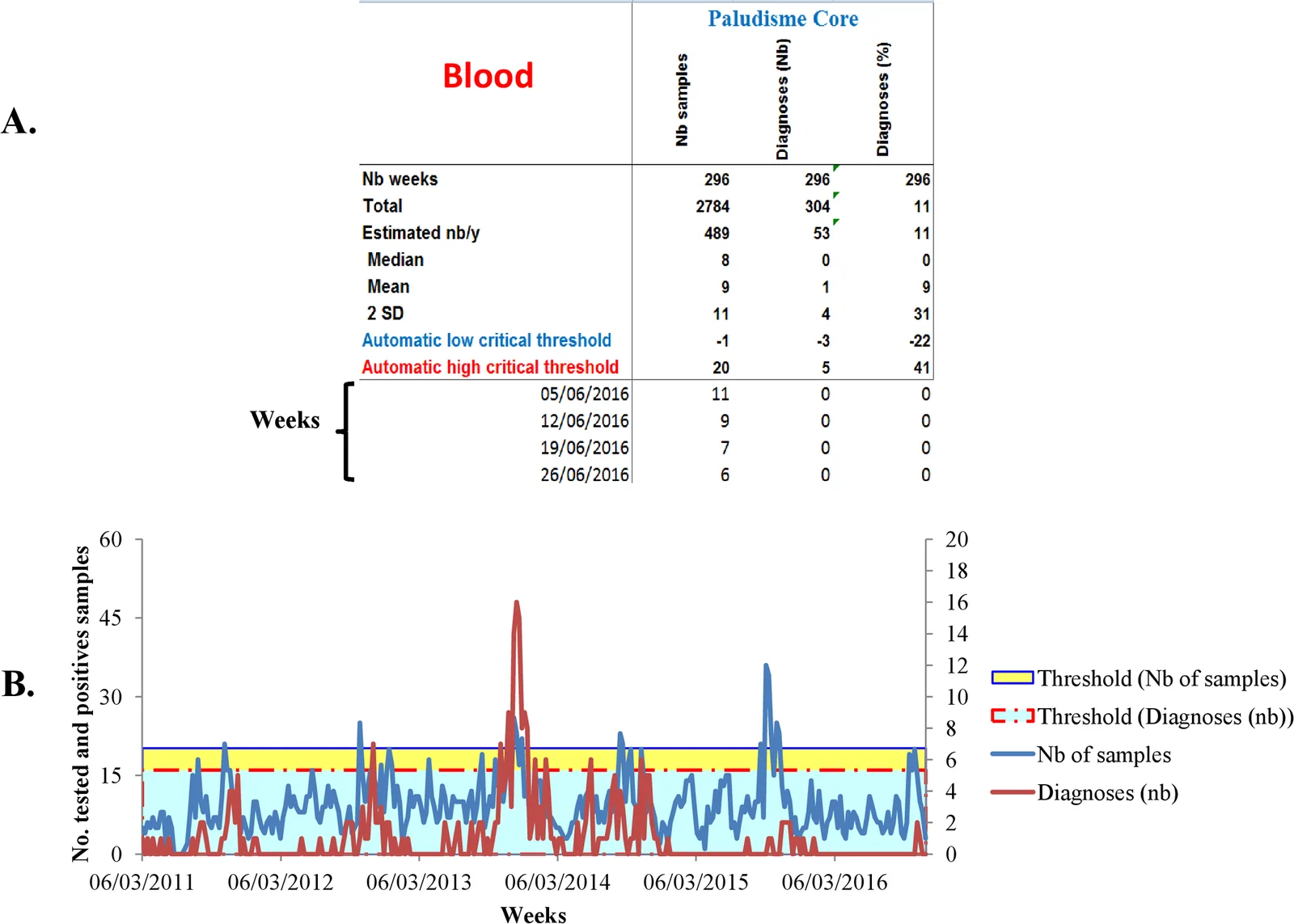
Example of epidemiological summary automatically produced by EPIMIC. Table A presents the weekly number of tested and positives samples automatically surveyed by the software. Plot B presents the historical trends of the number of tested and positives samples, and their historical thresholds.

Major improvements have been performed in the current EPIMIC version since its first description. All these improvements have been made using the Visual Basic programming language. The current version of EPIMIC consists in a single soft Microsoft Excel file which can be simply configured by following a step-by-step procedure proposed by the software during its first use. After this initial configuration, EPIMIC automatically collects and analyzes data from the different POC Microsoft Excel databases without any manual contributions, avoiding any inputs errors. If duplicates are present for the same week, the software only keeps the sample from which the most important number of positive tests have been identified. Moreover, the software automatically produces a Microsoft PowerPoint summary of the epidemiologic situation in the two POC-L using plots and screen shots when alarms are emitted for pathogens monitored by the software (Fig 2). In this way, data are anonymized to garanty the confidentiality of the patients’ data. Furthermore, the software separates the data according the place they come from (per villages for the Dielmo and Ndiop database, and per health structures for the Niakhar database), facilitating the weekly analysis of the epidemiological events. Finally, the software is able to rapidly produced a precised activity report summarizing the activity of the surveillance system for each of the POC-L.

### Analysis of the databases of the POC laboratories by EPIMIC

Once a week, the two Microsoft Excel databases are sent by the technicians to the coordinator of the epidemiological surveillance located in the Institut de Recherche pour le Développement (IRD) de Dakar. The databases are visually checked to avoid any inputs error. Then, EPIMIC is used to analyse the databases. The analysis is simple and rapid (less than 5 minutes per databases in routine).

### Analysis and investigations of the alarms emitted by EPIMIC, and sending of feedbacks

**Fig 3:**
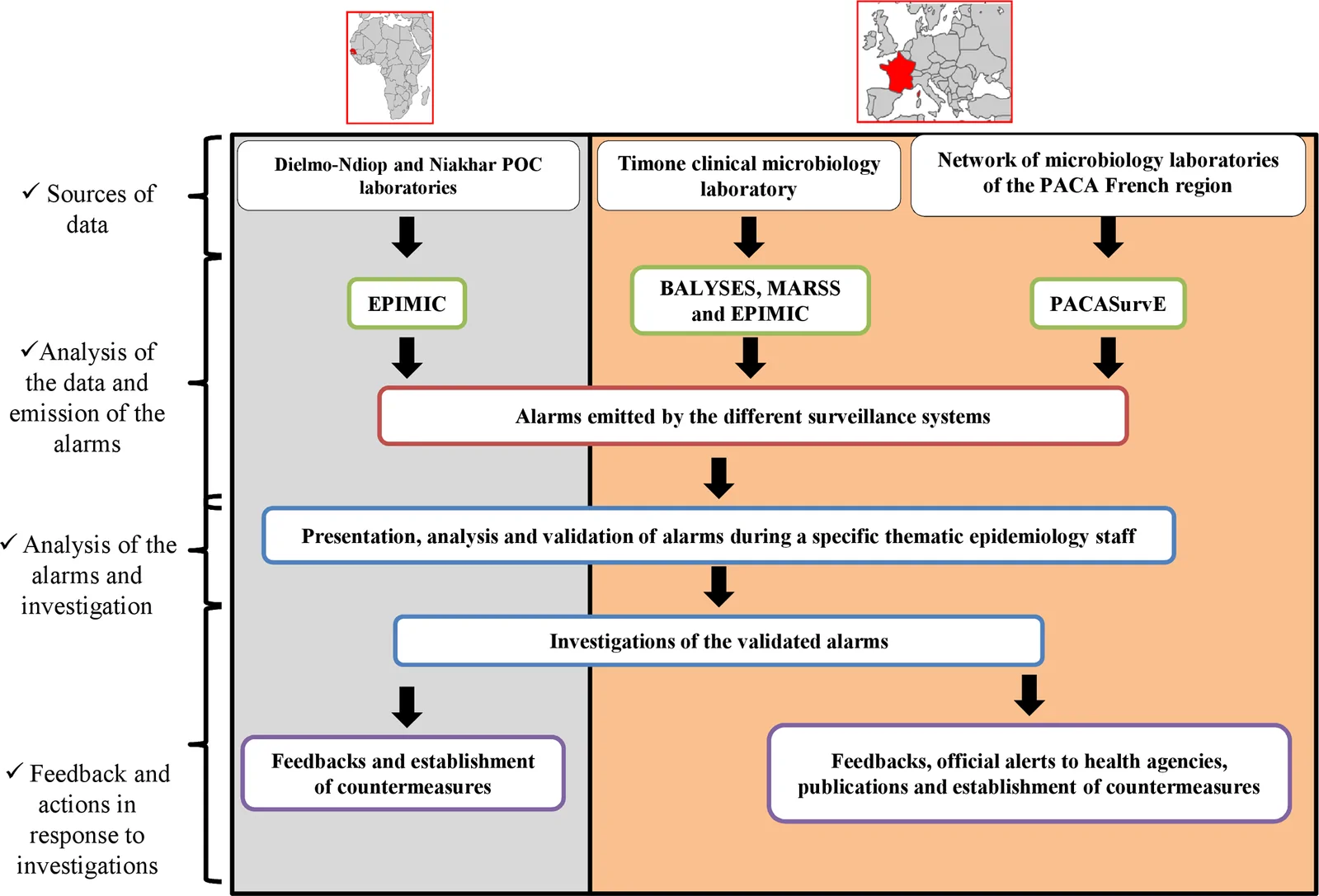
Workflow of the network of real-time surveillance systems implemented by the Institut Hospitalo-Universitaire Méditerranée Infection (Marseille, France) and the Institut de Recherche pour le Développement (Dakar, Senegal). POC: Point-Of-Care; PACA: Provence-Alpes-Côte d'Azur; EPIMIC: EPIdemiological surveillance and alert based on MICrobiological data; BALYSES: the Bacterial real-time Laboratory-based Surveillance System; MARSS: the Marseille Antibiotic Resistance Surveillance System; PACASurvE: the PACA Surveillance Epidemiological System.

Once analysed, the two Microsoft PowerPoint summaries are sent to a group of Senegalese and French specialists of the Institut Hospitalo-Universitaire Mediterranée Infections (IHU) of Marseille and of the Institut de Recherche pour le Développement (IRD) for analysis (Fig 3). If alarms are validated, indicating that the abnormal epidemiological event detected by the EPIMIC is possibly a true know or unknown epidemiological event, investigations are conducted to determine the veracity of the epidemiological events underlined by the alarms, and take countermeasures if necessary.

### Literature search on laboratory-based surveillance for infectious diseases in Africa

We performed a PubMed French-English literature search from 1990 to August 2016 in order to identify laboratory-based surveys for infectious diseases in Africa (S1 Table).

### Ethics statement

The project was initially approved by the Ministry of Health and Preventive Medicine of Senegal and the assembled village population in 1990. The project was ethically approved in 2000, 2005 and 2009 (identification number of the ethical and scientific evaluation: SEN21/09 and SEN37/09). Since that, the National Ethics Committee of Senegal has conducted three site visits, and ad-hoc research committees of the Ministry of Health and Preventive Medicine, the Dakar Pasteur Institute and the IRD provided recommendations to continue the project. Moreover, ethical approval is renewed on a yearly basis. After careful explanation of the goals of the project to the assembled village population, written informed consent was obtained after POC diagnosis from all of the adult residents in the study villages and from the guardians of children under 15 years of age.

## Results

Table 2 summarizes the data from the two POC-L databases and their EPIMIC analysis.

**Table 2:** Summary of the epidemiological surveillance systems EPIMIC implemented in Dielmo and Ndiop, and Niakhar, June 2016.

Global features		Sites	
		Dielmo and Ndiop	Niakhar
**POC laboratory**	Begin of the activity of the POC laboratory	February 2011	January 2016
	Number of years included in the historical database	5	0
	Number of weeks included in the historical database	278	26
	Number of patients included in the historical database	775	298
	Rounded weekly mean number of patients (standard deviation; range)	10 (5; 1-36)	12 (7; 3-26)
**EPIMIC**	Begin of the surveillance	April 2016	May 2016
	Number of deduplicated patients-samples couples surveyed by EPIMIC	2,577	316
	Number of POC tests surveyed by EPIMIC	27	27
	Number of pathogens surveyed by EPIMIC	12	12
	Number of positives deduplicated patients-samples couples surveyed by EPIMIC	739	56
	Number of cells filled in EPIMIC	16,743	1,563
	Number of alarms emitted by EPIMIC (before EPIMIC; since EPIMIC)	443 (373; 70)	14 (8; 6)
	Pathogen the most cited by alarms emitted by EPIMIC	*Plasmodium falciparum*	*Borrelia* spp.

### Dielmo and Ndiop

The full-time activity of the Dielmo and Ndiop POC laboratory started 5 years ago in February 2011, i.e 278 weeks ago [9]. Over this period, 2,577 deduplicated patients-samples couples from 775 different patients have been tested to look for the presence of at least one of the 15 causes of fever (*Plasmodium falciparum*, flu, dengue, *Coxiella burnetii*, *Leptospira* spp., *Bartonella* spp. (including *B*. *quintana*), *Tropheryma whipplei*, *Borrelia* spp., *Rickettsia* spp. (including *R*. *prowazekii*, *R*. *conorii*, *R*. *africae*, *R*. *felis*), *Salmonella* spp., *Streptococcus pneumoniae*, and *Staphylococcus aureus*) detected by the 27 POC tests routinely performed in the POC laboratory. The mean number of patients weekly tested was 10 patients (range: between 1 to 36 patients per week). 739 deduplicated patients-samples couples were found to be positive for at least one of the tested pathogens. The retrospective analysis of the Dielmo and Ndiop POC laboratory historical database with EPIMIC virtually allowed to identify 443 alarms, 373 before April 2016 and 70 since April 2016. *P*. *falciparum* was the pathogen the most cited by alarms emitted by EPIMIC (47 alarms, 2,635 tested samples, and 301 positive samples), followed by *R*. *felis* (43 alarms, 2,411 tested samples, and 21 positive samples), flu (39 alarms, 2,237 tested samples, and 135 positive samples), *C*. *burnetii* (35 alarms, 2,513 tested samples, and 14 positives), and *Borrelia* spp. (34 alarms, 2,577 tested samples, and 126 positives) (Table 2).

### Niakhar

The POC laboratory of Niakhar has been set up in January 2016, i.e 26 weeks ago. Over this period, this POC laboratory tested 316 deduplicated patients-samples couples collected from 298 different patients to look for the presence of the same 15 pathogens using the same POC tests as those mentioned above. On average, 12 patients were weekly tested (range: between 3 to 26 patients per week). These tests allowed to identify 56 deduplicated patients-samples couples positive to at least one of the tested pathogens. The retrospective analysis of the historical database of the Niakhar POC laboratory with EPIMIC virtually allowed to identify 14 alarms, 8 before May 2016 and 6 since May 2016. *Borrelia* spp. was the pathogen the most cited by alarms emitted by EPIMIC (4 alarms, 315 tested samples, and 36 positive samples), followed by *P*. *falciparum* (3 alarms, 316 tested samples, and 5 positive samples) (Table 2).

### PubMed literature search

The PubMed literature search initiated in this paper allowed us to identify 16 infectious diseases laboratory-based surveys and/or surveillance systems in Africa (S1 Table). Most of them (5, 31%) were implemented in South Africa, 3 (18.7%) in Egypt, and the 8 (50%) others in 8 different countries (1 (6.2%) in Burkina Faso, 1 in Kenya, 1 in Senegal, 1 in Sudan, 1 in Togo, 1 in Tunisia, 1 in Uganda, and 1 in Zimbabwe). Seven (43.8%) of these surveillance systems were totally devoted to the surveillance of bacteria, 3 (18.7%) to the surveillance of viruses, 2 to the surveillance of parasites (12.5%), and 1 (6.2%) to the surveillance of fungus.

## Discussion

EPIMIC was firstly implemented in the clinical microbiology laboratory of the Timone hospital, Marseille, France, in order to detect abnormal epidemiological events using data from both routine and POC-L [10]. Thereafter, three other laboratory-data based surveillance systems have been developed to complete this surveillance [11] (Fig 3). As EPIMIC was able to detect numerous true epidemiological events without heavy human and economic resources in Marseille [10], we decided to test if it was possible to effectively spread it in the West African country of Senegal using data routinely produced by rural POC-L (Figs 1 and 3).

The effective implementation of EPIMIC in rural Senegal was performed in less than one month, demonstrating the great scalability and versatility of the system. This is especially due to the fact that it was developed on Microsoft Excel, with low human and computer resources, using a simple algorithm for the detection of abnormal epidemiological events, and with no need for data specifically produced for surveillance as the software was specifically developed for syndromic surveillance [6,10]. However, this implementation required major improvements related to local needs. Thus, as mentioned before, the software was fully automated for the collection, anonymisation and analysis of the data, and for the production of a simple Microsoft PowerPoint activity report (Fig 2). This improvement considerably decrease the level of complexity to use the software including for people without in-depth computer skills, which is crucial to ensure its optimal long-term use [11]. Moreover, it includes a simple step-by-step easy procedure making it able to rapidly evolve with changes with no need to deeply modifiy it (for example the addition or removal of pathogens to be monitored by the system). Finally, EPIMIC is currently able to geographically localize the patients according to their villages or the health structures they visited, facilitating the on-site investigations of the validated abnormal events.

The local set-up of the software was strongly facilitated by the fact that the two POC-L have been implemented and routinely used from some time (Tables 1 and 2) with laboratory technicians trained to fill two preformatted Microsoft Excel databases, avoiding bad quality data and its consequence on syndromic surveillance [12]. Moreover, the two sites are regularly supplied with reagents necessary to perform the different POC tests, which is crucial for continuous detection and monitoring of pathogens [8,9]. Finally, they are equiped with Internet connexions, preventing disruptions in the weekly sending of microbiogical data.

**Fig 4:**
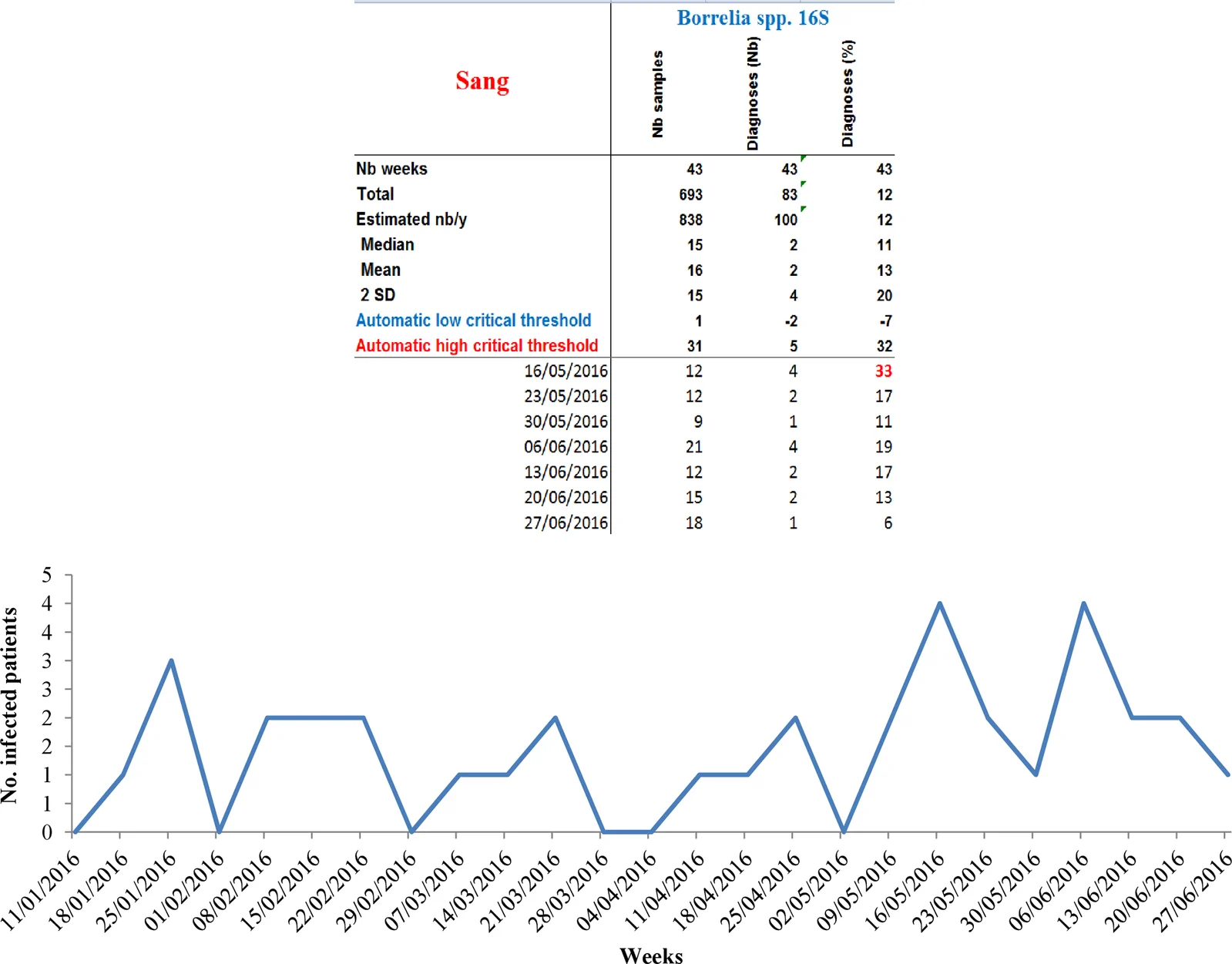
Epidemiological summary automatically produced by EPIMIC for *Borrelia* spp., Niakhar. The graphic presents the weekly evolution of the number of patients infected by the pathogen.

By the past, numerous studies have been performed in the Dielmo/Ndiop and Niakhar areas and they allowed us to identify that some emerging pathogens like *Coxiella burnetii*, *Bartonella* spp., *Tropheryma whipplei*, *Borrelia* spp., and *Rickettsia* spp were responsible for unexplained fevers in these areas [13–21]. However, these studies did not have for objective to monitor infections by these pathogens over periods in time in the area. This is now possible with the two EPIMIC surveillance systems. Thus, by counting the weekly number of patients infected by these emerging pathogens in the two rural areas under surveillance and by detecting abnormal events related to these pathogens, the EPIMIC systems allowed us to collect and analyze the information that will be used to improve our knowledge on the local epidemiology of the pathogens of interest, including the emerging ones. In this way, the fact that *Borrelia* spp. has been identified to be the pathogen the most cited by alarms triggered by EPIMIC from January 2016 to June 2016 in Niakhar (Fig 4) is already an unexpected information. Moreover, EPIMIC also provides data on the weekly number and proportion of infections that remain unexplained. These points make us strongly believe that these two surveillance systems will be rapidly of great interest locally for the detection and management of infectious diseases, including to implement and evaluate the onsite impacts of pre-existing and future prevention plans.

Comparing our surveillance with other surveillance implemented in Africa over the two last decades (S1 Table), we identified that the main asset of EPIMIC is the number of POC tests and pathogens it currently allows to survey in near real-time (27 POC tests for 15 pathogens (bacteria, viruses and parasites)). Nevertheless, further improvements are needed to enhance our EPIMIC syndromic surveillance network. Indeed, as mentioned before, EPIMIC has been implemented using the Microsoft Excel software, and abnormal events are currently detected using statistical tools based on the historical mean of the data +/- two standard deviations. Although this strategy allows the rapid development of the system and was valuable in terms of human and economic resources [11], this is not suitable for the monitoring of big databases including data with seasonal variations in pathogen isolation like flu and *P*. *falciparum*, or in case of rare pathogens. These issues are planed to be addressed with the development of a local web-based plateform including more sophisticated statistical tools for the accurate monitoring of abnormal events [22–24]. We also plan to integrate local Health Agencies to our surveillance network (Fig 3) in order to actively participate to the Senegal national surveillance of infectious diseases like it is the case in France [11].

To conclude, EPIMIC has been successfully spread using data routinely produced by two rural POC-L in Senegal, West Africa. We are clearly convinced that such initiatives, like the implementation of rural POC-L [13,20], are needed to improve our global knowledges on infectious diseases, whatever the level of knowledge we currently have on them.

## Supporting Information

S1 Table Summary of the surveillance systems implemented in Africa from 1990 to August 2016. (XLSX)
