# Correction: Implementation of syndromic surveillance systems in two rural villages in Senegal

**DOI:** 10.1371/journal.pntd.0014748

**Published:** 2026-09-28

**Authors:** The PLOS Neglected Tropical Diseases Editors

## Notice of Republication

This Correction supersedes and resolves the previously published Expression of Concern for the linked article [1,2].

Following the publication of the article and Expression of Concern [1,2], PLOS investigated concerns pertaining to the reported ethical approval and the article’s adherence to *PLOS Neglected Tropical Diseases*’ research ethics policies.

Specifically, the study reports the use of patient medical data collected at Point-Of-Care (POC) laboratories in Dielmo/Ndiop and Niakhar, Senegal, to develop an automated pathogen surveillance system. The article states that medical data was accessed both retrospectively and prospectively, and that the retrospective analysis of the Dielmo and Ndiop POC laboratory historical database with EPIMIC identified 373 alarms before April 2016, and 70 alarms since April 2016. The ethics statement reports that the projects with evaluation numbers SEN21/09 and SEN37/09 received ethics approval in 2000, 2005, and 2009, the Institut de Recherche pour le Développement de Dakar recommended continuation of the project, and ethics approval was renewed on a yearly basis.

A representative of the Aix-Marseille Université Ethics Committee stated that the implementation of EPIMIC in the rural Senegalese villages took place in 2016, and that the data used to evaluate its implementation were collected between April and May 2016. They clarified that patient identifiable information was only accessible to the clinicians and laboratory technicians working at the POCs, and that the researchers received anonymized data sets only. They also indicated that the EPIMIC work reported in the PLOS article did not require ethics approval, as it involved application of an automated tool to data collected within an existing health surveillance program that was covered under continuous ethical oversight by Senegalese authorities.

The representative provided copies of the documents listed below for editorial review.

- Ethics approval document N°00.86 MSP/DS/CNERS (issued on June 2, 2010, valid for one year) by the Ministère de la Santé et de la Prévention of the République de Sénégal for protocol SEN21/09 titled *“Identification des agents pathogènes responsibles de fièvre au Sénégal. Réalisation de tests diagnostiques chez les malades consultant dans les dispensaires de Dielmo et Ndiop.”*
- Ethics approval document N°00.87 MSP/DS/CNERS (issued on June 2, 2010, valid for one year) by the Ministère de la Santé et de la Prévention of the République de Sénégal for protocol SEN37/09 titled *“Identification des agents pathogènes responsibles de fièvre au Sénégal (projet IDEPATH) dans les sites suivants: Niakhar (Fatick), Mlomp (Ziguinchor), Banafassi (Kédougou) et Keur Momar Sarr(Louga).”*
- Document N°001380/MSP/DS/DER (issued May 31, 2011) by the Ministère de la Santé et de la Prévention of the République de Sénégal. The document provides administrative authorization to implement protocols SEN21/09 and SEN37/09 for one year.
- Document N°00081/MSAS/DGS/DS/CNERS (issued June 4, 2012) by the Ministère de la Santé et de l’Action Sociale of the République de Sénégal, provides continued approval for the protocols SEN21/09 and SEN37/09.

Based on the documentation and the information received, PLOS concluded that the study meets the journal’s human research ethics requirements.

This article was republished on September 10, 2026, to replace the map presented in Fig 1 with an image that is available under a CC BY 4.0 license. In addition, Table 2 was updated to rectify a formatting error. Please download this article again to view the correct version.
